# Direct observation of microcavitation in underwater adhesion of mushroom-shaped adhesive microstructure

**DOI:** 10.3762/bjnano.5.103

**Published:** 2014-06-25

**Authors:** Lars Heepe, Alexander E Kovalev, Stanislav N Gorb

**Affiliations:** 1 Functional Morphology and Biomechanics, Zoological Institute, Kiel University, Am Botanischen Garten 1-9, 24118 Kiel, Germany

**Keywords:** bio-inspired, biomimetic, cavitation, contact mechanics, gecko, interface, negative pressure, pull-off, surface, tribology

## Abstract

In this work we report on experiments aimed at testing the cavitation hypothesis [Varenberg, M.; Gorb, S. *J. R. Soc., Interface* **2008,** *5,* 383–385] proposed to explain the strong underwater adhesion of mushroom-shaped adhesive microstructures (MSAMSs). For this purpose, we measured the pull-off forces of individual MSAMSs by detaching them from a glass substrate under different wetting conditions and simultaneously video recording the detachment behavior at very high temporal resolution (54,000–100,000 fps). Although microcavitation was observed during the detachment of individual MSAMSs, which was a consequence of water inclusions present at the glass–MSAMS contact interface subjected to negative pressure (tension), the pull-off forces were consistently lower, around 50%, of those measured under ambient conditions. This result supports the assumption that the recently observed strong underwater adhesion of MSAMS is due to an air layer between individual MSAMSs [Kizilkan, E.; Heepe, L.; Gorb, S. N. Underwater adhesion of mushroom-shaped adhesive microstructure: An air-entrapment effect. In *Biological and biomimetic adhesives: Challenges and opportunities*; Santos, R.; Aldred, N.; Gorb, S. N.; Flammang, P., Eds.; The Royal Society of Chemistry: Cambridge, U.K., 2013; pp 65–71] rather than by cavitation. These results obtained due to the high-speed visualisation of the contact behavior at nanoscale-confined interfaces allow for a microscopic understanding of the underwater adhesion of MSAMSs and may aid in further development of artificial adhesive microstructures for applications in predominantly liquid environments.

## Introduction

During the past two decades, bio-inspired microstructured adhesives became a new class of adhesive materials with different potential applications (e.g., in robotic systems, medicine, and industrial pick-and-place processes), due to the reversible and residue-free character of the sticking mechanism [1–4]. So far, the most promising candidates for technical applications are surface microstructures with mushroom-shaped contact geometry (see review [4]), which have been studied intensively under various loads (e.g., preload [5–6], shear [7–8], overload [9], and tilt [10–11]) and environmental conditions (e.g., dry in air [5–12], oil lubricated [13], on rough substrates [13–15], in vacuum [10,16–18], and underwater [19–20]). The origin of the high adhesion capability of the mushroom-shaped adhesive microstructures (MSAMSs) was attributed to the combination of intermolecular van der Waals forces and a particular failure mode at detachment, a consequence of an optimized homogeneous stress distribution in the contact interface during pull-off [21–22]: During detachment of an individual MSAMS from a substrate, a crack nucleates somewhere in the middle of the contact area and further propagates towards the outer edge while the perimeter remains still in contact until complete separation occurs. This type of failure mode, further called mode II [21], suggests the formation of a low-pressure zone in the contact area giving rise to a suction effect [9]. However, such effect at a dry interface was shown to contribute only marginally (at most ≈10%) to the overall measured pull-off forces [10,16–18].

In their recent work Varenberg and Gorb [19] have observed that the pull-off forces measured underwater were significantly higher (about 25%) compared to those measured under ambient conditions. This effect cannot be explained by intermolecular van der Waals forces. The authors hypothesized that the enhanced underwater adhesion may be a result of cavitation under each individual MSAMS when entrapped water in the contact area is subjected to a negative pressure (tension) during pull-off and the liquid water turns into vapor at a certain cavitation threshold [19]. This effect would make each individual MSAMS to act as a passive suction device [19].

In the present study, we report on underwater adhesion experiments with individual MSAMSs. The visualisation of the MSAMS interface using the combination of high speed video recording and reflection contrast microscopy under applied pull-off force aimed at testing the cavitation hypothesis, an effect that have never been experimentally observed in artificial bio-inspired microstructured adhesives.

## Experimental

### Experimental setup

In the experiments, two individual MSAMSs, denoted by sample 1 and sample 2, were detached from a smooth glass slide under different wetting conditions with simultaneous video recording of the failure dynamics with a setup similar to a reflection interference contrast microscope (RICM) [23–24]. Individual MSAMSs were cut off from the microstructured tape made from polyvinylsiloxane (PVS) with a thickness of the supporting polymer film of about 900 µm [5,11,22]. Pull-off forces were measured using a force measuring system (FMS) consisting of a tensometric force transducers FORT-10 (World Precision Instruments, Inc., Sarasota, Florida) fixed on a three-axis micromanipulator F-131.3SS (Physik Instrumente GmbH & Co. KG, Karlsruhe, Germany) [11]. The FMS was installed on an inverse light microscope Observer.A1 (Carl Zeiss MicroImaging GmbH, Göttingen, Germany) equipped with a “Plan-Neofluar 63×/1.25 Antiflex” oil-immersion objective (Carl Zeiss MicroImaging GmbH). The microscope was operated in the epi-illumination mode and the complete spectrum of the light source HXP 120 (Carl Zeiss MicroImaging GmbH) was used. Detachment behavior was recorded with an attached high-speed camera Photron Fastcam SA1.1 (VKT Video Kommunikation GmbH, Pfullingen, Germany) either with 54,000 frames/s or with 100,000 frames/s. Obtained high-speed video sequences were background corrected by using the average of at least 10 frames at the end of the sequences where MSAMSs were already detached.

**Figure 1:**
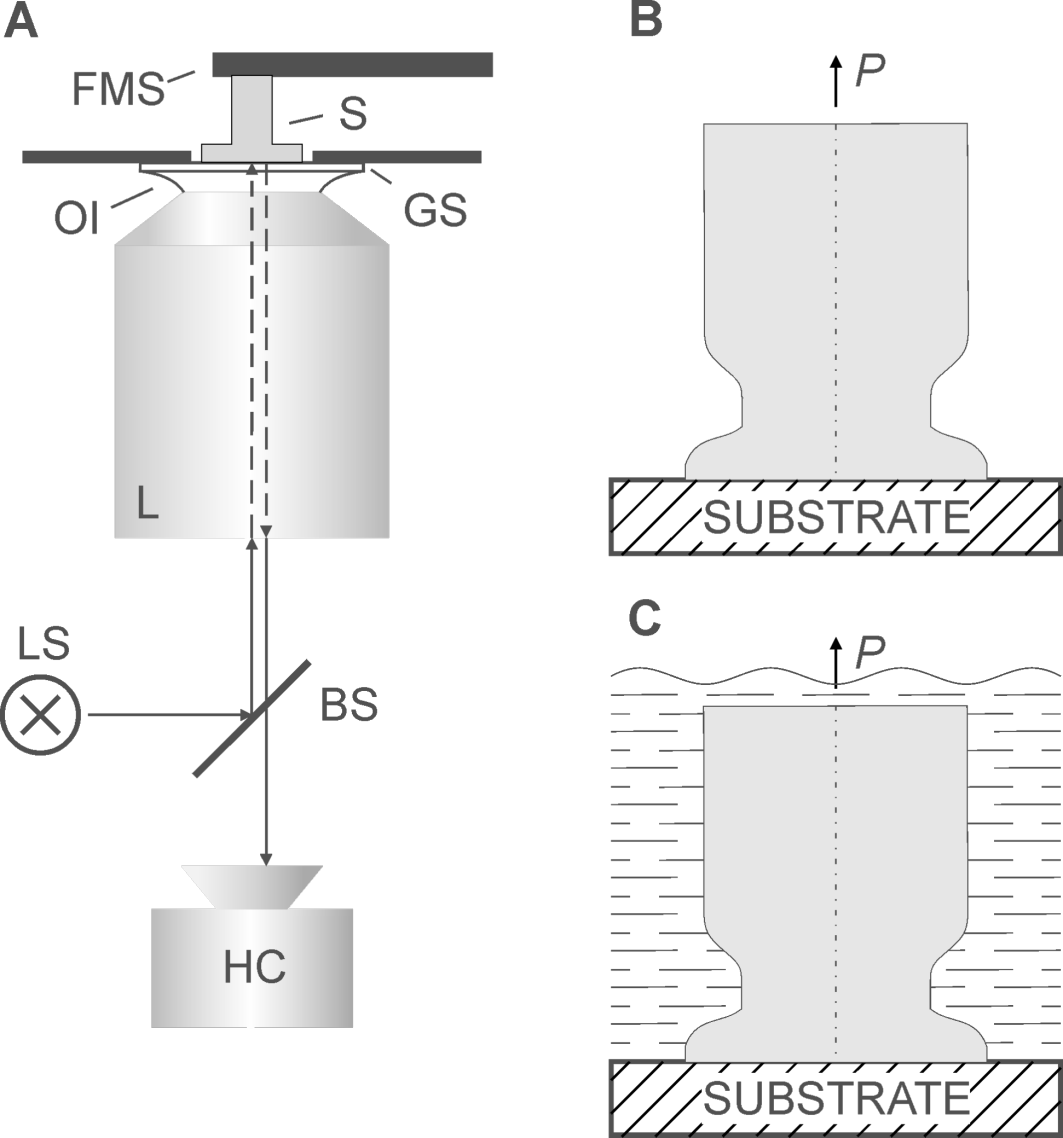
Schematic of the experimental setup (A). FMS, force measuring system; S, sample; GS, glass slide; OI, oil immersion; L, lens; LS, light source; BS, beam splitter; HC, high-speed camera. Individual MSAMS in contact with a substrate under ambient conditions (B) and submerged in water (C). *P* is the applied force.

Figure 1A shows the schematic of the experimental setup. In order to repeatedly attach and detach samples, individual MSAMSs were glued to the force transducer. To ensure parallel alignment between samples and the glass slide, first individual MSAMSs were attached manually to the glass slide using tweezers while observing the proper contact via the microscope. Then, attached to the glass slide, samples were withdrawn at a retraction velocity of 10 µm/s in the direction normal to the surface of the glass slide. In order to test the cavitation hypothesis, pull-off forces were measured at detachment on individual MSAMS samples under different wetting conditions. For sample 1, the following measurement sequence was performed: 1. Pull-off forces were measured at ambient conditions, further called 'dry state' (Figure 1B). 2. After reattachment, i.e., contact formation in dry state, pull-off force was measured with the sample 1 submerged in water by applying a drop of deionized water onto the individual MSAMS with a syringe (Figure 1C), further called 'dry–wet' state. 3. Then, pull-off forces were measured after reattachment of the individual MSAMS under submerged conditions, further called 'wet' state (Figure 1C). For sample 2, only the dry state and the wet state could be compared.

### Image formation and simulation

**Figure 2:**
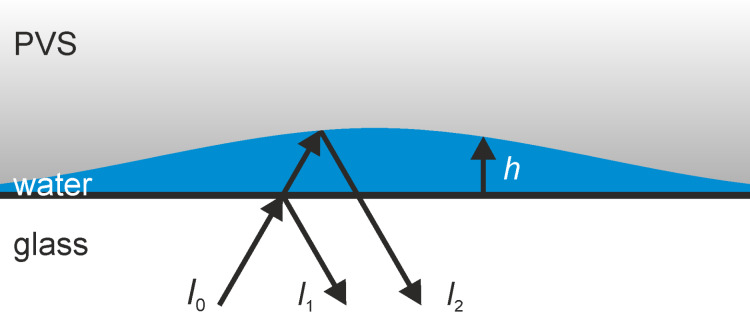
Schematic of the optical path in the RICM at the glass–water–PVS layers. The incoming beam with intensity *I*_0_ is partially reflected at the glass-water interface (*I*_1_) and at the water–PVS interface (*I*_2_). The optical path difference between *I*_1_ and *I*_2_ depends on the distance *h* between glass and PVS, the angle of incidence, and on the refractive index of water.

In order to reliably interpret the high-speed video sequences of the underwater detachment behavior of individual MSAMSs, we simulated the image formation obtained in the RICM experiments according to the theory described in [25]. Consider the case, when an individual MSAMS, submerged in water and partially detached from the glass substrate, where the detached regions are filled with water (Figure 2), is observed in epi-illumination as depicted in Figure 1A. Then, the incoming light with intensity *I*_0_ is partially reflected at the glass–water interface (*I*_1_) and superimposes with the reflected light *I*_2_ from the water–PVS interface (Figure 2). Depending on the degree of coherence, roughly a measure of the ability to interfere, which is defined by the mutual coherence function Γ_12_, the total reflected intensity *I* can be written in its general form

[1]
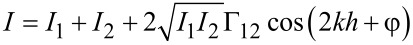


where k = 2π*n*_water_/λ is the wave vector with *n* being the refractive index of the medium (here water) and λ the wavelength. The constant phase φ accounts for potential phase shifts of π at reflectance at an optical denser medium. For Γ_12_ = 0 (incoherent case), beam 1 and beam 2 cannot interfere and Equation 1 is reduced to *I* = *I*_1_ + *I*_2_. Γ_12_ > 0 corresponds to a partial coherence and Γ_12_ = 1 describes the fully coherent case. According to the van Cittert–Zernike theorem Γ_12_ is [25]

[2]
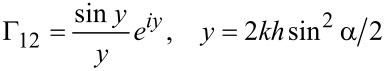


where α is the maximum illumination angle. Substituting Equation 2 in Equation 1 yields, according to Rädler and Sackmann [25]

[3]
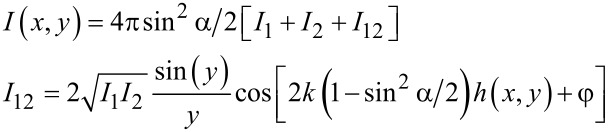


here *h*(*x*,*y*) denotes the distance between glass and PVS surface along the *x* and *y* direction. Since *n*_PVS_ > *n*_water_ (see below) with *n*_PVS_ and *n*_water_ being the refractive index of PVS and water, respectively, beam *I*_2_ undergoes a phase shift of π at the water–PVS interface (Figure 2). The intensities *I*_1_ and *I*_2_ can be calculated by

[4]
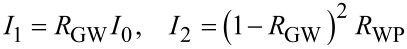


where *R*_GW_ and *R*_WP_ denote the reflectance at the glass–water and water–PVS interface, respectively. In general, the reflectance at an interface of medium 1 to medium 2 can be calculated from Fresnel's equations and takes, in the simplest case of normal incidence, the form [25]

**Figure 3:**
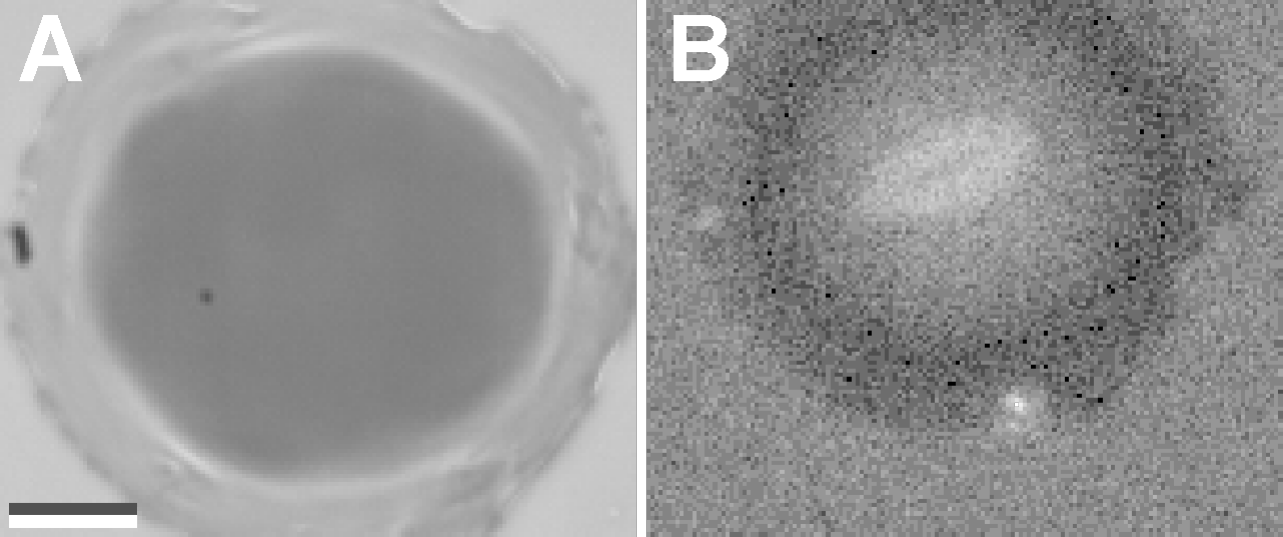
Sample 1 in dry contact with glass (A) and in contact with glass underwater (B). Scale bar, 10 µm.

[5]
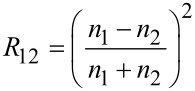


with *n*_1,2_ being the refractive indices of medium 1 and 2. Since *n*_PVS_ is unknown we first determined the refractive index using the image A of Figure 3 where sample 1 was in dry contact with the glass. Incoming intensity *I_0_* was determined from the background using Equation 2 and Equation 3 at the glass-air interface. For numerical values of the refractive indices of glass and air see Table 1. Since no interference pattern is visible in the region under the thin contact plate (i.e., Γ_12_ = 0) and assuming intimate contact (i.e. glass-PVS interface) the total reflected intensity in that region is simply the sum *I* = *I*_1_ + *I*_2_, whereas *I*_2_ is the second reflection at the PVS-air interface. The refractive index *n*_PVS_ was adjusted to match the measured intensity in that region. We found *n*_PVS_ = 1.468 to be a good estimate which is well in the range of refractive indices of silicon rubber [26]. Next we used the image B of Figure 3 to determine again *I*_0_, but now for the underwater case. For simplicity, the distance *h*(*x*,*y*) between the glass and PVS surface was modelled Gaussian-like by

[6]
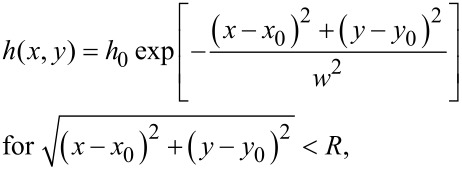


where *R* is the radius of an individual MSAMS, (*x*_0_, *y*_0_) the origin of the circular contact of an individual MSAMS, *w* the width of the distribution, and *h*_0_ the maximum height. Using Equation 3 and a reasonable set of parameter (see Table 1) a still image of the detachment of an individual MSAMS was simulated in order to interpret the experimentally obtained detachment sequences. Results were rounded to greyscale values between 0 (black) and 255 (white) in accordance to the 8-bit output of the high-speed camera. Finally, shot noise was added to the simulated image so that the standard deviation of the background is comparable to the noise obtained from Figure 3B.

**Table 1:** Parameter and its values used in the simulation.

parameter	value

grid size, *N* × *N*	150 × 150
refractive index of air, *n*_air_	1.000
refractive index of water, *n*_water_	1.335
refractive index of glass, *n*_glass_	1.526
refractive index of PVS, *n*_PVS_	1.468
incoming light intensity, *I*_0_	10462
light wavelength, λ	550 nm
radius of MSAMS, *R*	(N – 10)/2
width of distribution, *w*	0.375*R*
maximum height, *h*_0_	150 nm
constant phase, φ	π
maximum illumination angle, α	25°

## Results and Discussion

**Figure 4:**
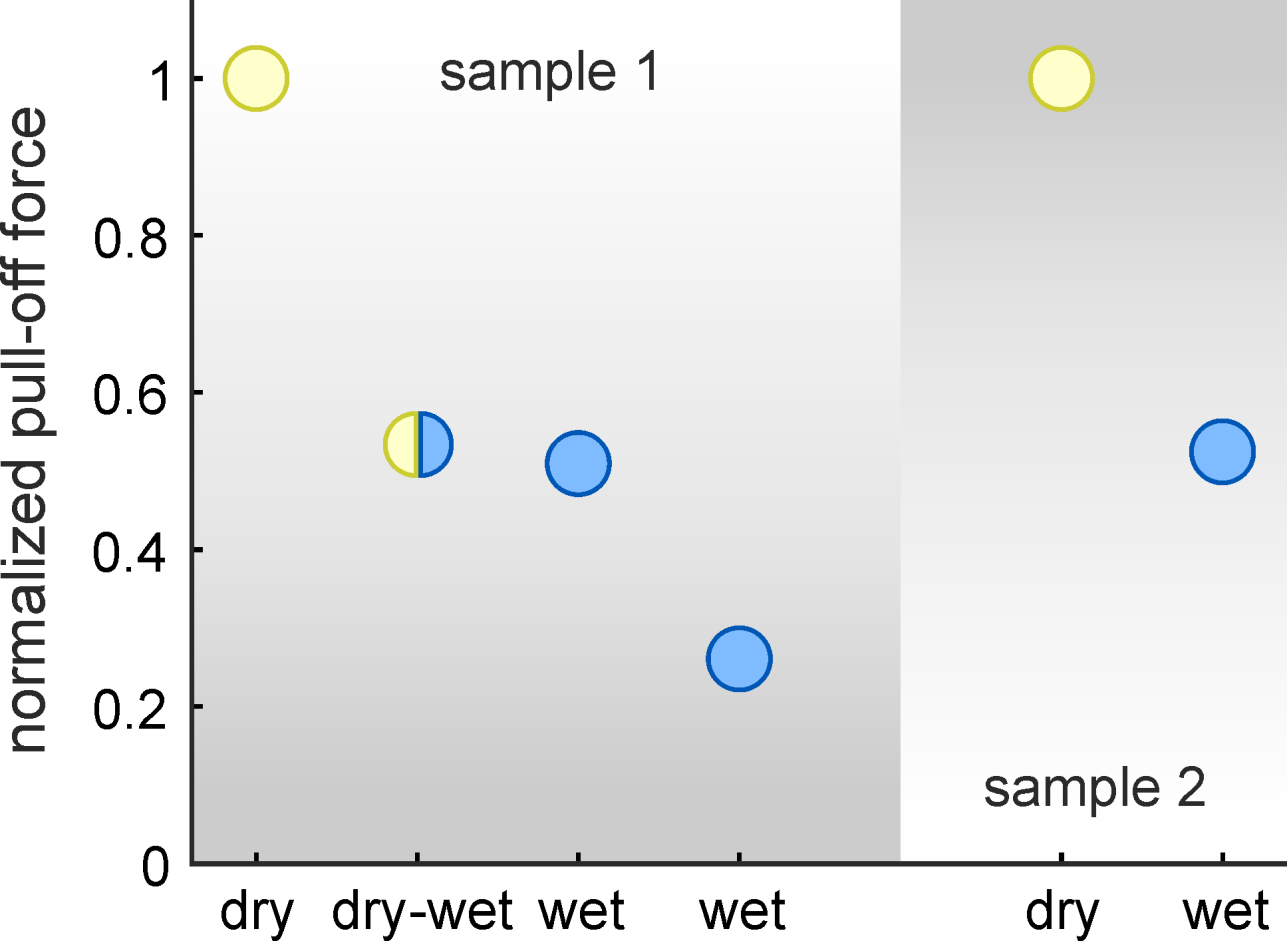
Pull-off forces, normalized by the average value of the dry state. Two individual MSAMS samples were measured on glass substrates for different wetting conditions: dry state (yellow circle), dry–wet state (yellow/blue circle), and wet state (blue circles). Dry state corresponds to measurements under ambient conditions. Dry–wet state corresponds to measurements where the sample has been brought into contact under dry conditions, but subsequently submerged in water. Wet state corresponds to measurements where the sample has been brought into contact when already submerged. For sample 1 the median dry state pull-off force was ca. 570 µN (*N* = 5, min. value ca. 540 µN, and max. value ca. 590 µN). For sample 2 the median dry state pull-off force was ca. 490 µN (*N* = 5, min. value ca. 440 µN, and max. value ca. 570 µN).

We measured pull-off forces of individual MSAMSs on glass substrates under different wetting conditions (Figure 4). Pull-off forces were normalized with respect to those obtained in the dry state. Dry state pull-off forces were averaged over five individual measurements for both samples. For the sample 1, the median dry state pull-off force was ca. 570 µN (*N* = 5, min. value ca. 540 µN, and max. value ca. 590 µN). For the sample 2, the median dry state pull-off force was ca. 490 µN (*N* = 5, min. value ca. 440 µN, and max. value ca. 570 µN). In the dry–wet state, the pull-off force of sample 1 was about 55% of the dry state. In the wet state, the pull-off forces for both samples were both about 50% of the dry state, except for the second measurement of sample 1 in the wet state, which was about 25%. These values are in agreement with recent macroscopic adhesion measurements on arrays of MSAMSs completely wetted by water and submerged underwater [20]. For the first classification of these results we may consider the following two limiting cases in the underwater adhesion of MSAMS. In the first case, a thin water layer separates the glass–MSAMS contact. Then the van der Waals interaction strength, described by the Hamaker constant between glass and MSAMS, is expected to be reduced by about 86% [19]. In the second case, MSAMS and glass form a dry contact underwater. In this case, one would expect similar pull-off forces as observed in the dry state. However, the experimentally obtained results lie somewhere in between these two cases.

**Figure 5:**
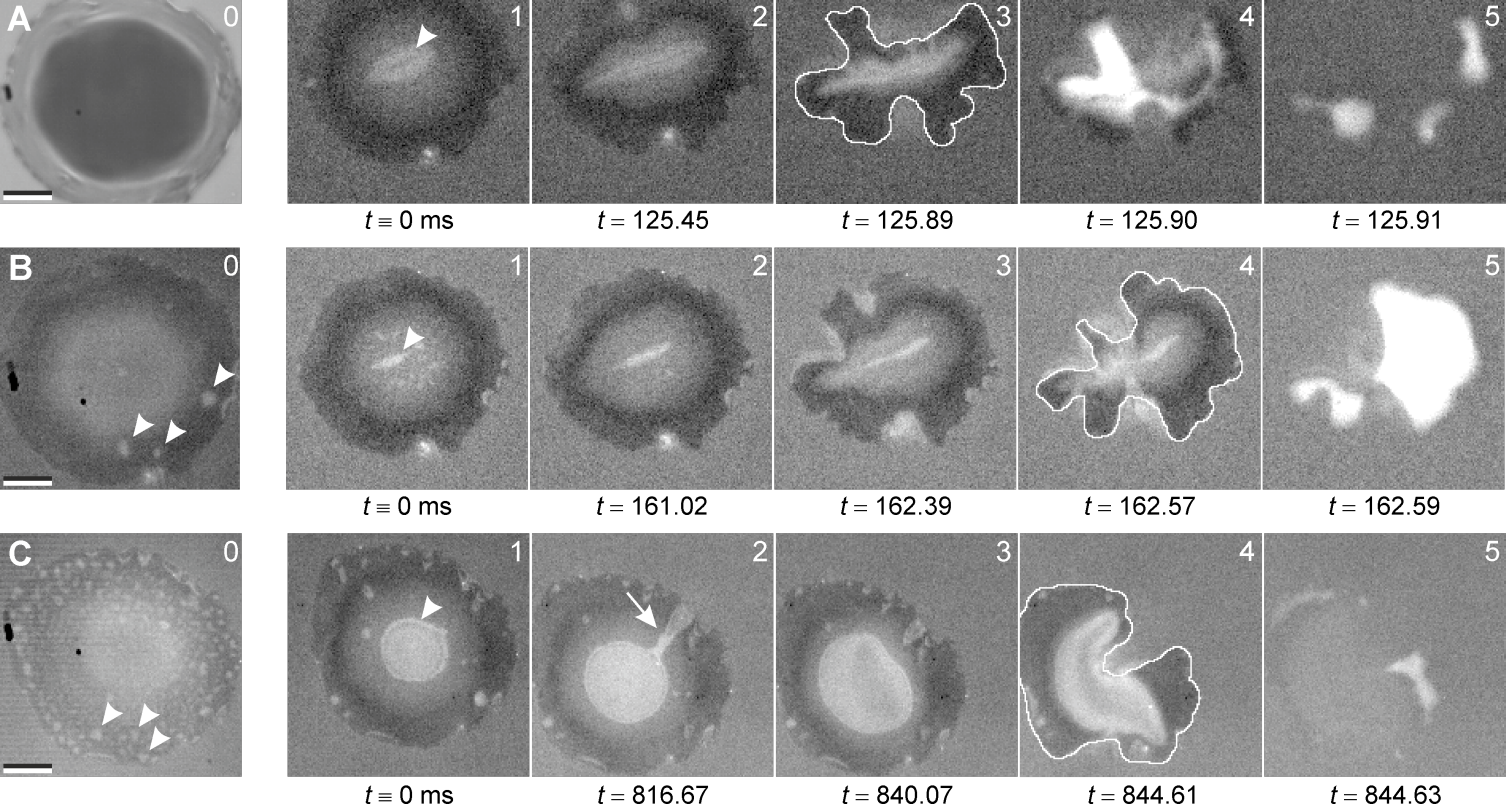
Detachment sequences of individual MSAMSs separating from a glass substrate for the sample 1 in the dry–wet state (A), second measurement of the sample 1 in the wet state (B), and the sample 2 in the wet state (C). Images labelled with '0' correspond to equilibrium conditions right after contact formation. Note, in (A) contact formation was under ambient conditions. Images with index '1' to '5' are still images of the actual detachment sequence. Frames set to *t* ≡ 0 ms (images with index '1') were arbitrarily chosen as a reference. All times are given in milliseconds (ms). During detachment water inclusions were present within the contact interface of MSAMS and glass (white arrowheads). In sequences (A) and (B) microcavitation was observed indicated by the white region in images A4–5 and B5. The white arrow (C2) indicates channel formation by interfacial crack propagation between the confined water within the contact interface and the surrounding water. The white traces in A3, B4, and C4 outline the contact area prior to detachment. For comparison, detachment sequences in the dry state are shown [4,9,11,17]. Scale bar, 10 µm.

For each measurement also the detachment behavior was video recorded, in order to observe the actual failure process of the MSAMS detachment from the glass substrate. Figure 5 shows detachment sequences in the dry–wet state (A, sample 1) and in the wet states (B, sample 1 second measurement; C, sample 2). Images labelled with '0' correspond to equilibrium conditions right after the contact formation. Images with index '1' to '5' are still images of the actual detachment sequence. Frames set to *t* ≡ 0 ms (images with index '1') were arbitrarily chosen as a reference. For better comparability all images have the same scale. For comparison, detachment sequences in the dry state are shown in [4,9,11,17].

**Figure 6:**
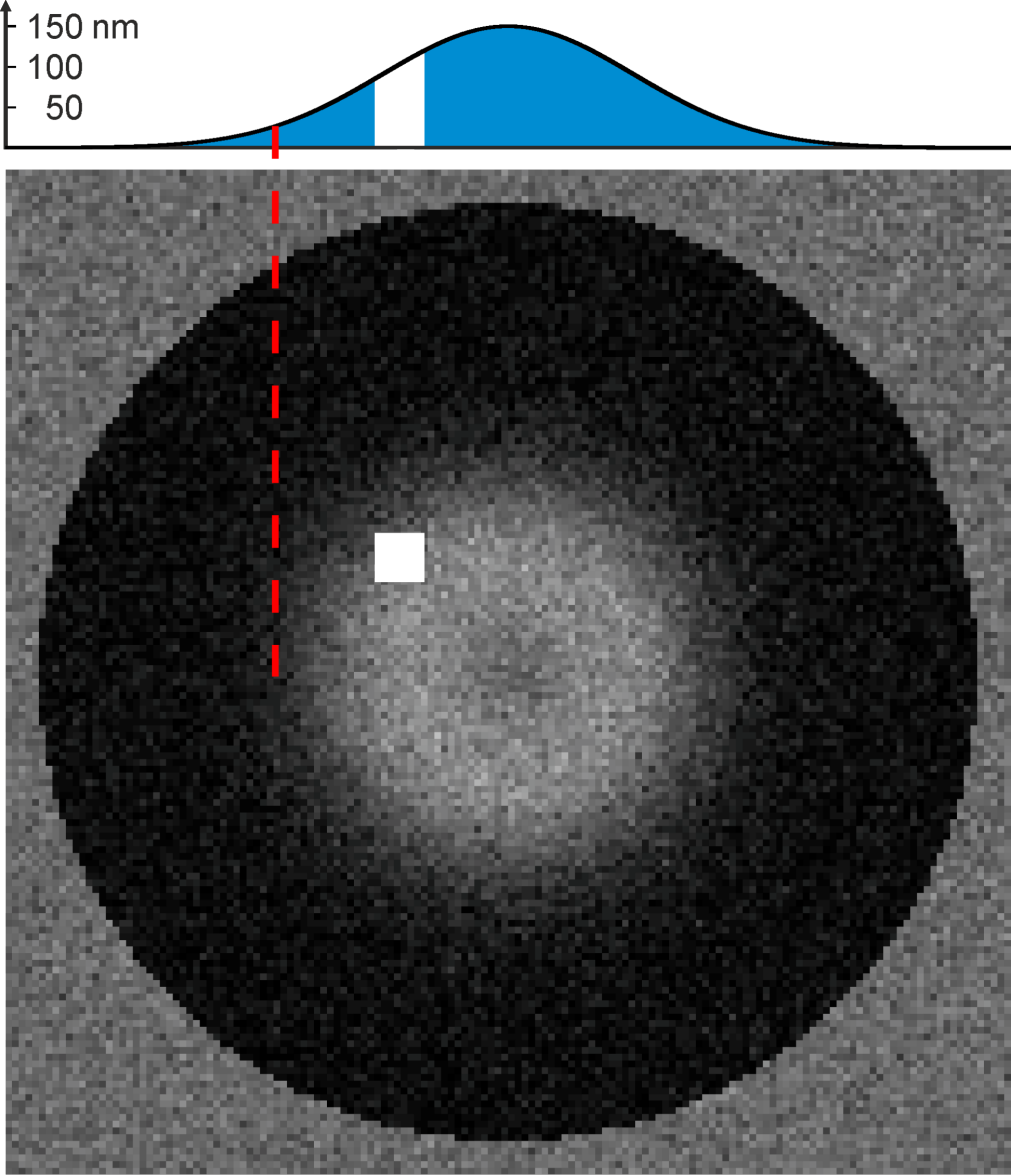
Simulated image of an individual MSAMS partially detached from a glass substrate submerged in water, for which the gap filled with liquid water was, for simplicity, chosen Gaussian-like with a maximum separation of 150 nm in the middle of the contact (see graph at top of the simulated image). The simulation parameters are given in Table 1. Note that around the maximum separation in the middle of the contact the lower greyscale values (darker region) indicate a first order interference minimum (zero order minimum corresponds to intimate contact) similar to what is observed in Figure 5A1. The red dashed line indicates separation of about 25 nm. Due to the noise level even in the simulation it is not possible to reliably distinguish between contact and non-contact region for a separation below 25 nm. The white square has been calculated assuming the gap being filled with air instead of water.

Let us first observe that during detachment, also in the dry–wet state (Figure 5A), water inclusions were present within the contact interface of MSAMS and glass (see white arrow heads in Figure 5). In order to confirm this observation we simulated an individual MSAMS partially detached from a glass substrate with the gap between the MSAMS and glass being filled with liquid water, not with air (for details see Experimental section: Image formation and simulation). The gap was, for simplicity, simulated as a Gaussian-like shape with its maximum separation of 150 nm in the middle of the contact. The result is shown in Figure 6. The light grey central region, which indicates the non-contact state, is clearly visible. Around the maximum separation in the middle of the contact the lower greyscale values (darker region) indicate a first-order interference minimum (zero order minimum corresponds to intimate contact) similar to what is observed in Figure 5A1. The red line in Figure 6 indicates the position with a separation between glass and MSAMS of about 25 nm. Note that with the particular noise level, heights below 25 nm cannot be clearly resolved even in the simulation. Thus, in that range, a contact region cannot be reliably distinguished from a non-contact region. The white square in Figure 6 was calculated assuming the gap being filled with air instead of water. We are thus very confident that we indeed observed water inclusions within the contact interface of MSAMSs and glass. This is particular interesting, especially in the case of dry contact formation (dry–wet state), since MSAMSs (hydrophobic) and glass (hydrophilic) form a Janus interface in which confined water may exhibit non-trivial behavior [27]. However, in a recent experiment it has been confirmed that water can "leak" into a (dry) Janus interface formed by glass and MSAMSs [28]. During the further detachment process the contact area shrank to about 60% (Figure 5A) of the initial contact area (images labelled with '0') without losing contact. Then, at the very moment of detachment a sudden (within less than 20 µs) and significant change in contrast is observed (see white areas especially in Figure 5 A4–5 and B5). This dramatic change in contrast clearly indicates cavitation.

We can calculate the pull-off strength σ for all sequences dividing the obtained pull-off forces *P* by the contact area *A*_det_ right before complete separation and cavitation (area within white counters in Figure 5 A3, B4, C4). We obtain σ_A_ ≈ 430 kPa, σ_B_ ≈ 180 kPa, and σ_C_ ≈ 260 kPa for sequences A–C, respectively. By using *P* = Δ*pA*_det_ = (*p*_atm_ – *p*_1_)*A*_det_ with *p*_atm_ the atmospheric pressure and *p*_1_ the pressure in the liquid, we estimate negative pressures subjected to the confined water of about −0.33 MPa, −0.08 MPa, and −0.16 MPa, for sequences A–C, respectively. These values are well in the range observed for mechanically stretched water and may indicate heterogeneous nucleation at gas residues (see [29–30]). One may also consider the role of the fluid viscosity, which may strongly affect the fluid motion [31] during contact formation and breakage between MSAMS and substrate. This may result in a viscous contribution to the observed pull-off stress. However, in a recent publication [17] the effect of a suction contribution to the adhesion of MSAMS was tested by comparing pull-off forces obtained at atmospheric and reduced pressure at different retraction velocities. It was shown that at a sufficiently low retraction velocity (100 µm/s) no suction contribution was observed [17]. At higher velocities (400 µm/s and 800 µm/s) a suction effect of about 10% contributed to the overall pull-off force [17]. This was explained by air being able to percolate through the contact interface (due to, e.g., surface roughness resulting in partial contact) and instantaneously balancing the pressure in the forming low pressure zone in the center of the contact area [17]. For water, the viscosity of which is much larger than the viscosity of air, such a 'critical' retraction velocity will be shifted towards lower values. We thus assume that the fluid volume which enters the contact interface during detachment from outside is very small and trapped water in the contact interface is effectively sealed from the outer environment.

Finally, it is important to mention a particularly interesting observation in sequence C (Figure 5C). During detachment the large amount of small water inclusions (Figure 5C0) accumulated to a large non-contact region in the middle of the contact area (Figure 5C1). At a certain load a channel formed (white arrow in Figure 5C2) by interfacial crack propagation balancing (at least partially) the pressure between the confined water within the contact interface and the surrounding water. Interestingly, the channel closed again before a complete separation occurred (Figure 5C3). Although not observed under dry conditions such channel formation may also be an additional explanation why at low retraction velocities no suction effect was found in the dry adhesion of MSAMS arrays [17].

## Conclusion

We confirmed the cavitation hypothesis proposed in [19] in the underwater adhesion of individual MSAMSs. We found underwater pull-off forces consistently lower, approximately 50%, of those under ambient conditions which cannot be explained by one of the two limiting cases: (1) MSAMS and glass form a dry contact underwater, (2) MSAMS and glass are separated by (thin) water layer. Instead we observed that water inclusions present at the interface are subjected to negative pressure (tension) during applied pull-off. However, for individual MSAMSs used in this work, we did not observe higher underwater adhesion, when compared to the dry state as reported in [19]. This supports the assumption that the observed enhanced underwater adhesion reported in [19] is probably an effect of the air retaining properties of MSAMS arrays, when submerged underwater [20]. Our results allow a microscopic understanding of the underwater adhesion of MSAMSs and may aid in further development of artificial adhesive microstructures for applications especially in liquid dominated environments.
